# Healthcare Students’ Perceptions and Attitudes Towards Peers with Autism Spectrum Disorders

**DOI:** 10.1007/s10803-024-06368-5

**Published:** 2024-05-03

**Authors:** Vasiliki Zarokanellou, Evridiki Papagiannopoulou, Alexandros Gryparis, Vassiliki Siafaka, Dionysios Tafiadis, Vassiliki Ntre, Nafsika Ziavra

**Affiliations:** 1 Department of Speech and Language Therapy, School of Health Sciences, University of Ioannina, 4th Km National Road Ioannina, Athens, Ioannina, 45500 Greece; 2 Department of Nursing, School of Health Sciences, University of Ioannina, Ioannina, Greece; 3 Department of Nursing, “Aghia Sophia” Children’s Hospital, Athens, Greece

**Keywords:** Autism spectrum disorder, Attitudes, Perceptions, Postsecondary education, Autism label, Peers

## Abstract

**Purpose:**

The present study investigates healthcare students’ affective, behavioral, and cognitive attitudes toward hypothetical peers with autism spectrum disorder (ASD) and the effect of the ASD label on their attitudes.

**Methods:**

The MAS scale for ASD persons in the postsecondary education (Matthews et al., 2015) was translated and adapted in Greek according to the guidelines of World Health Organization (2016). Participants (*n* = 444) were randomly divided into three equal groups and completed their demographic information. Each participant read the three vignettes of the Greek-adapted MAS scale describing a communicative interaction with one hypothetical fellow student with autistic characteristics in three different social situations but in each group, the vignette’s character was labeled differently (High-functioning, typical college student, and no-label).

**Results:**

Students in the High-functioning group demonstrated more positive affective, behavioral, and cognitive attitudes toward the vignette characters than students in the no-label condition. Furthermore, students in the typical student group reported more rejective behaviors toward the vignette characters than students in the high-functioning group, implying that atypical behavior evokes rejection and stigmatization, while the label did not. Male students presented more positive cognitive attitudes across study groups in comparison to female students, while previous contact with individuals with ASD did not seem to impact significantly attitudes.

**Conclusion:**

The findings of the study indicate that knowledge of an ASD diagnosis leads to greater acceptance and have important implications for future research, disability policy makers, and university support services.

Autism Spectrum Disorder (ASD) is a lifelong neurodevelopmental disorder that affects approximately one in 100 children globally (Zeidan et al., 2022). It is characterized by persistent deficits in social communication and social interaction across multiple contexts, as well as restricted and repetitive patterns of behavior (American Psychiatric Association, 2013). In Greece, recent data indicate an overall national prevalence between 0.94% and 1.15% for children until the age of 17 (Kouznetsov et al., 2023; Thomaidis et al., 2020), which is similar to the referred worldwide estimates. The latest epidemiological studies for ASD suggest that most people with ASD (66.7%) present normal intelligence and attend regular school settings (Zeidan et al., 2022). There is a research gap around the postsecondary educational experiences of autistic people, however, there are fair indices that the number of intelligence-capable individuals with ASD enrolling in higher education (HE) is growing and will increase more in the future (Bakker et al., 2023; Matthews et al., 2014). A Pan-American study reported that 2% of registered students with disability in HE programs have ASD (Raue & Levis, 2011). A similar study in the UK revealed that 0.45% and 0.17% of HE enrollments in Northern Ireland and the rest of the United Kingdom respectively were students diagnosed with ASD (Karola et al., 2016). Finally, a longitudinal study in the Netherlands recorded that from 2010 to 2016 the percentage of students with ASD who enrolled at a Dutch major University increased significantly from 0.20 to 0.45% (Bakker et al., 2023). In Greece, until today there is no data about this issue, but a very recent legal adjustment in 2022 (Law 6069 (issue B) /28-11-2022) permits autistic people to enter University without taking exams, thus making one believe that the percentage of students with ASD will increase in Greek Universities onwards.

## Characteristics of ASD Students in Higher Education

Because of their unique cognitive style which is the ability to focus on details and memorize with ease, creative thinking skills, and a tendency to acquire accurate knowledge, which can lead to passionate interests, academic pursuits are likely to be appealing to ASD students (Bakker et al., 2023). However, the scarce research that has examined their transition in HE claims that ASD students experience both academic and non-academic challenges (Bakker et al., 2023; Gurbuz et al., 2019; Petcu et al., 2021; Hees et al., 2015) which may lead them to academic failure and personal breakdown, even though they have the intellectual potential to succeed. Compared to other students with disability, ASD students present higher rates of comorbidity, with the most common conditions being dyslexia, ADD/ADHD, and mental health disorders such as depression and anxiety disorders (Bakker et al., 2023; VanBergeijk et al., 2008). Furthermore, ASD students may struggle with sensory overload in and out of campus, which may trigger an emotional breakdown (tantrum or meltdown), as well as stereotypies (Bakker et al., 2023; Cai & Richdale, 2016; Hees et al., 2015). Moreover, they present poor daily living skills and show significant deficits in interpersonal interactions, which may lead to social isolation (Bakker et al., 2023; Cai & Richdale, 2016; VanBergeijk et al., 2008; Hees et al., 2015). The above can jeopardize their social adjustment, make their everyday living difficult, and eventually result in dropping out of their studies (Bakker et al., 2023, Lei et al., 2020; Hees et al., 2015). ASD students face also significant academic barriers, which include poor communication skills, difficulties in understanding abstract or ambiguous concepts, difficulties in processing different information quickly, deficiencies in organizational skills, and time management, as well as deficits in managing social demands linked with the course, group work or presentations (Bakker et al., 2023; Gurbuz et al., 2019; Hees et al., 2015). These academic barriers pose important pressure on ASD students, affecting seriously their academic performance and their personal well-being.

## The Impact of Label, Peer Acceptance, and Autism Awareness on the Successful Inclusion of ASD University Students

There is a growing literature investigating peers’ openness toward their ASD classmates and how their level of knowledge about autism modulates their attitudes, as peers’ acceptance plays a crucial role in ASD students’ academic and social inclusion (Butler & Gillis, 2011; Gardiner & Iarocci, 2014; White et al., 2016). Due to the fact that ASD is often an invisible impairment, students with autism may go unnoticed until they decide to reveal their diagnosis, which several of them deny disclosing for fear of being stigmatized (White et al., 2016). Butler and Gillis (2011) revealed that ASD behaviors resulted in stigmatization, not the label itself. Another study found that knowledge of the diagnosis may improve cognitive and behavioral attitudes from peers toward ASD persons e.g. allowing peers to be more positive toward the socio-communicative deficits of ASD persons (Matthews et al., 2015). A cross-cultural study revealed that autism awareness may relate positively to decreased misconceptions and stigma toward ASD persons and increased knowledge about ASD (Obeid et al., 2015). Generally, studies indicate that university students are quite knowledgeable about ASD (Gardiner & Iarocci, 2014; Obeid et al., 2015; Tipton & Blacher, 2014; White et al., 2016), but higher knowledge about ASD is not always related to more positive attitudes towards ASD students, suggesting that attitudes are more resistant to change (Gardiner & Iarocci, 2014; Matthews et al., 2015; White et al., 2016). Factors such as gender, culture, academic direction, and quantity and quality of previous interpersonal contact experiences with ASD persons impact openness toward them (Gardiner & Iarocci, 2014; Matthews et al., 2015; Obeid et al., 2015; White et al., 2016) although overall ASD peers are accepted in terms of more distant relationships (Gardiner & Iarocci, 2014). In particular, previous direct and positive interactions with ASD persons lead to higher openness toward ASD fellow students (Gardiner & Iarocci, 2014; White et al., 2016), while mixed results have been reported about gender (Butler & Gillis, 2011; Gardiner & Iarocci, 2014; Lu et al., 2020; Matthews et al., 2015; Obeid et al., 2015; White et al., 2016) with most studies supporting that gender has a small influence on the attitudes towards ASD classmates.

## Measurements on Attitudes Towards ASD People

There are only two scales measuring attitudes toward ASD people. The first one is the Societal Attitudes Toward Autism (SATA) Scale (Flood et al., 2013) and the second is the Multidimensional Attitude Scale Toward Persons with Disabilities (MAS) (Findler et al., 2007). MAS (Findler et al., 2007) is a 34-item scale, that examines attitudes towards people with disabilities. In Greek, it has been validated showing sound psychometric properties (Govina et al., 2020). Several studies have explored its factor structure (Lu et al., 2020). The original scale is aligned with the 3-factor approach, which claims that attitudes are constructs with affective, cognitive, and behavioral components (Findler et al., 2007), however, several adapted versions support different 4-factor and 5-factor models which mostly segment the component “affect” into a negative and a positive sub-construct. The scale has also been adapted for ASD people in French (Dachez et al., 2015), Chinese (Lu et al., 2020), and Japanese (Tsujita et al., 2021), while recently it was modified (Park et al., 2023) to investigate attitudes toward people with different types of disabilities, namely anxiety disorder, ASD, blindness, and schizophrenia. Finally, Matthews, and colleagues (2015), adapted the vignettes of MAS to reflect common social situations in postsecondary education for assessing peer attitudes toward college students with ASD. Measurement experts on attitudes suggest that multidimensional scales such as MAS and indirect methods of measurement where participants are aware that they are being measured but have only dim clues about the measurement situation (projective technique) are preferred over unidimensional and direct methods of measurement as they show better psychometric properties (Antonak & Livneh, 2000; Findler et al., 2007). Direct methods may have a considerable bias since respondents may alter or distort their answers to be more socially acceptable (Antonak & Livneh, 2000; Findler et al., 2007).

## Purpose of the Study

The estimation of the predominant attitudes of neurotypical students concerning ASD peers is necessary for suggesting desired ends to policymakers and designing effective programs to modify attitudes toward possible ASD fellow students (Antonak & Livneh, 2000). In Greece, however, there is scarce research (Veroni, 2019; Papadopoulos, 2021; Papadopoulos et al., 2022; Zarokanellou et al., 2023) regarding the attitudes toward people who are on the spectrum of autism. The adapted MAS scale for ASD persons is one of the two instruments that have been used for studying attitudes towards ASD persons. Relevant research strongly recommends the updating and refining of multidimensional scales that differentiate between the components of attitude (affects, cognition, behaviors) and the investigation of the relationship between each of these components and other variables such as gender and social contexts (Findler et al., 2007). The purpose of the current study is twofold. Firstly, to translate and adapt the MAS scale in Greek for assessing attitudes towards people with ASD and assess its psychometric properties, and secondly to investigate the perceptions and attitudes of Greek undergraduate healthcare students about cognitively able individuals with ASD. Healthcare students are the future healthcare professionals, a population who are perceived to be knowledgeable and show positive perceptions and attitudes toward ASD people (Bania et al., 2021). This positive attitude plays a major role in the quality of treatment and intervention services provided to these individuals.

## Methods

### Participants

Data were collected from a convenience sample of 444 undergraduate (Mean Age: 21.2 years, SD = 4.4 years) healthcare students. All participants were recruited from the Departments of Nursing and Speech-Language Therapy of the University of Ioannina, Greece, and had agreed to participate voluntarily in the study. The sample was imbalanced in gender proportion (most of the participants were women, ≈ 82%) reflecting the demographics of students at these two Departments (Table 1).

**Table 1:** Descriptive statistics of participants’ sociodemographic characteristics, by study group

Parameters	No Label Group (A)	Typical University Student Group (B)	HFA Label Group (C)	*p*-value*
**Gender N(**%**)**				
Male	20 (13.5)	19 (12.6)	17 (11.7)	0.914
Female	120 (81.1)	126 (83.4)	118 (81.4)	
NA	8 (5.4)	6 (4.0)	10 (6.9)	
Total number	148 (33.3)	151 (34.0)	145 (32.7)	
**Year of study N(**%**)**				
1	39 (26.4)	41 (27.2)	40 (27.6)	0.794
2	45 (30.4)	45 (29.8)	44 (30.3)	
3	25 (16.9)	28 (18.5)	19 (13.1)	
4	26 (17.6)	23 (15.2)	21 (14.5)	
5	13 (8.7)	14 (9.3)	21 (14.5)	
**Relationship with an autistic person N(**%**)**				
No relationship	115 (77.7)	121 (80.1)	122 (84.1)	0.383
I have a relative/friend	21 (14.2)	24 (15.9)	22 (15.2)	
I have a family member	6 (4.1)	3 (2.0)	1 (0.7)	
NA	6 (4.1)	3 (2.0)	0 (0.0)	

HFA: High-Functioning Autism; NA: no answer

*: p-value from Pearson’s chi-square test

### Procedure

Prior to data collection, the study’s protocol was approved by the Research and Ethics Committee of the University of Ioannina (RN: 49,627) and a research use license for the translation and adaptation of the measurements was acquired by the authors of the measurements. Examinees were credited one extra point as a bonus for their participation in the study, regardless of whether they completed all measurements. The study was conducted in two research phases. Before the administration of the instruments, students were informed broadly about the scope of the study, reporting that the study is to investigate how people behave in different social situations. In the first phase of the study, participants completed their demographic characteristics and were randomly assigned to one of three study groups to complete the MAS scale. Like in the study of Matthews et al. (2015), groups differed only by the statements describing the main character of each vignette. The first group (Group A) was the no-label group (*n* = 148); the second group (Group B) was the typical university student label condition (*n* = 151), and the third group (Group C) was the high-functioning ASD label condition (*n* = 145). Specifically, participants in groups A, B, and C read the following statements:

1. Group A: No-label (Name of the main character) is in the same year in university as you and is of above-average intelligence.
2. Group B: Typical University student label (Name of the main character) is in the same year in university as you and is a typical university student of above-average intelligence.
3. Group C: HFA label (Name of the main character) is in the same year in university as you, has high-functioning autism, and is of above-average intelligence. Individuals with high-functioning autism sometimes demonstrate difficulty with social interaction and appropriate communication. However, most are of average to above-average intelligence.

Each participant assigned to one of the three groups read three vignettes describing a communicative interaction with three different characters who displayed characteristics commonly observed in ASD people, such as restricted interests, difficulties in social interaction, and a need for sameness. These social interactions depicted common social situations for university students such as a group assignment in a class project, joining a voluntary group, or a shared living situation.

After reading the vignettes, participants had to rate their emotions, thoughts, and attitudes toward the vignette characters.

In the second phase of the study, which was conducted after a week, participants received through their academic email a link and a notification, which was politely asking them to complete the second phase of the research. The link led to a Google form, which displayed the Greek translation and adaptation of the SATA scale (Zarokanellou et al., 2023). In this phase, they were written informed that the study was examining attitudes toward people with high-functioning ASD and were asked to proceed and complete the form. 214 participants responded and completed the Greek SATA scale (Zarokanellou et al., 2023), participating in the second phase of the research.

### Measurements

#### Multidimensional Attitudes Scale Toward Persons with Disabilities (MAS, Findler et al., 2007)

This scale is a self-report 34-item 5-point Likert instrument for the measurement of attitudes toward individuals with disabilities which is coded such that a higher score indicates more negative attitudes toward ASD persons. The questionnaire is divided into three subscales, namely affects (16 items), cognitions (10 items), and behaviors (8 items). In the current study, the adapted vignettes of the study of Matthews et al., (2015), for assessing peer attitudes toward ASD college students were used for the same purposes. For the translation and adaptation of the vignettes of the MAS scale, a forward and backward translation procedure took place, as indicated by the World Health Organization (Robine & Jagger, 2003). Two members of the research team, who are proficient bilingual speakers of Greek and English translated independently into Greek the adapted MAS scale (Matthews et al., 2015) and then discussed and agreed on a reconciled Greek version of the test. Then, the Greek version of the instrument was back-translated into English by an independent bilingual professional translator and the back-translation was reviewed, establishing the pre-final version of the questionnaire. Finally, the comprehensibility of the questionnaire was checked by asking ten students to review the items of the questionnaire and minor modifications were made, leading to the final version of the questionnaire.

#### The Greek Version of the Societal Attitudes Toward Autism (SATA) Scale (Zarokanellou et al., 2023)

The SATA scale (Flood et al., 2013) is a 26-item 4-point Likert standardized scale that assesses attitudes toward people with ASD. It includes 3 subscales which evaluate: (a) personal attitudes towards people with ASD (16 items, range score 16–64) (b) knowledge about the disorder (5 items, range score 5–20), and (c) Personal Distance which refers to the extent to which participants are willing to interact with individuals with ASD across situations (5 items, range score 5–20). According to the 4-point Likert system, 1 point declares strong disagreement with the statement, while 4 points declare strong agreement. Overall, lower total scores on the SATA scale reflect better knowledge and more positive attitudes regarding ASD persons. The questionnaire is standardized for the Greek language demonstrating acceptable psychometric properties with an overall Cronbach’s alpha value equal to 0.872 and Cronbach alpha values over 0.76 for each of the three subscales (Societal Attitudes: a = 0.812, Personal Distance: a = 0.765, Knowledge: a = 0.843) (Zarokanellou et al., 2023).

### Data Analysis

Categorical variables are presented as absolute and relative (%) frequencies. Quantitative variables are presented as mean ± SD. For the statistical analysis, reverse coding for SATA and MAS scales was used as proposed in the original versions of the two tools (Findler et al., 2007; Flood et al., 2013). Missing data were managed using multiple imputation, as suggested by Rubin (1987). Multiple imputation provides a method for dealing with missing data by using statistical methods to create multiple sets of plausible values for the missing data based on the observed data. By creating these multiple sets, this technique properly accounts for the uncertainty associated with missing data. In our case, missing values were replaced by plausible values in each of five simulated datasets using the *mice* library in R statistical software.

To examine the internal consistency and reliability of the MAS scale Cronbach’s alpha coefficient was used, and the analysis was performed for each of the subscales, and overall. Next, Spearman’s correlation coefficient was implemented to evaluate the linear relationship between different MAS subscales. Lastly, to assess the concurrent validity of this version of the MAS scale, the correlations between MAS and SATA questionnaire responses were calculated, using Spearman’s correlation coefficient.

For the main analysis, a preliminary 3 (Group) X 3 (vignette) repeated measures analyses of variance (ANOVAs) was used to compare significant differences in scores from each MAS subscale among the three vignettes in the full sample and within each study condition (reported in Table 2). Finally, a hierarchical regression analysis was implemented to examine the effect of group (HFA label, typical student label, and no-label), familiarity with a person with ASD, and gender on each MAS subscale (affective, cognitive, and behavioral). The study condition entered always in the first step of the regression analysis, followed by the familiarity with a person with ASD in the second step. In the last step, gender was entered into the regression analysis.

A two-tailed p-value < 0.05 was considered statistically significant. Statistical analysis was implemented using ΙΒΜ SPSS v. 28 (IBM Corp. Released 2021. IBM SPSS Statistics for Windows, Version 28.0. Armonk, NY: IBM Corp.) and (RStudio Team (2020). RStudio: Integrated Development for R. RStudio, PBC, Boston, MA URL http://www.rstudio.com/).

## Results

### Reliability and Validity of the Adapted Greek MAS Scale for ASD

The internal consistency of this Greek version of the MAS scale was excellent. Specifically, Cronbach’s alpha coefficient was equal to (a) 0.827 for the Affects subscale, (b) 0.879 for the Cognitions subscale and (c) 0.800 for the Behaviors subscale. Overall, Cronbach’s alpha was equal to 0.889 (95 C.I. %: 0.878–0.898).

Spearman’s correlation coefficient between the MAS and the SATA scale’s total scores was − 0.155 (*p* = 0.024) (Table 2). 214 participants completed adequately the Greek version of the SATA scale (Zarokanellou et al.,^2023^).

**Table 2:** Spearman correlation coefficient between MAS subscales and SATA total scores

MAS subscales	Correlation coefficient	*p*-value
Affects	-0.140	0.041*
Cognitions	-0.046	0.507
Behaviors	-0.154	0.025*
Overall total score	-0.155	0.024*

*p < 0.05

The statistical analysis showed a significant, negative, association between all subscales of MAS except Cognitions subscale with the SATA total score, as well as between the MAS total score and the SATA total score (Note here that a higher score on the MAS scale reflects more negative attitudes towards people with ASD, while for the SATA questionnaire, the opposite is true).

## Differences between Groups and Social Situations (Vignettes)

Results from the 3 × 3 repeated measures ANOVA revealed significant differences in scores in each MAS subscale among the three vignettes in the full sample and within each study Group (reported in Table 3). The no-label group had significantly higher total scores on affects, behaviors, and cognitions subscales in comparison to the HFA label group (*p* < 0.05). For the total sample of participants, the Mean for cognitions was found to be the significantly (*p* < 0.001) highest (M = 3.35, SD = 0.79), followed by affects (M = 2.45, SD = 0.53) and behaviors (M = 2.18, SD = 0.74), indicating that participants showed the most negative attitudes toward an ASD person on the cognitive dimension and the least negative on the behavioral component.

**Table 3:** Descriptive statistics: MAS subscale scores by vignette and study group

	Total sample (Ν = 444) M (SD)	HFA label (Ν = 145) M (SD)	Typical label (Ν = 151) M (SD)	No label (Ν = 148) M (SD)
**MAS total scores**	2.66 (0.86)	2.57 (0.85)	2.63 (0.84)	2.78 (0.87)
**Affects total scores**	2.45 (0.53)	2.40 (0.53)	2.43 (0.50)	2.52 (0.56) ^c^
Group project (Kyle)	2.40 (0.46)	2.38 (0.47)	2.38 (0.43)	2.44 (0.47)
Club meeting (Tim)	2.27 (0.53) ^a^	2.21 (0.52) ^a^	2.25 (0.50) ^a^	2.35 (0.58)
Shared living space (Alex)	2.69 (0.52) ^ab^	2.62 (0.52) ^ab^	2.66 (0.49) ^ab^	2.76 (0.54) ^ab^
**Behaviors total scores**	2.18 (0.74)	2.05 (0.70)	2.15 (0.71) ^c^	2.35 (0.78) ^c^
Group project (Kyle)	1.92 (0.58)	1.77 (0.52)	1.89 (0.55)	2.09 (0.61)
Club meeting (Tim)	2.35 (0.79) ^a^	2.14 (0.72) ^a^	2.35 (0.80) ^a^	2.56 (0.80) ^a^
Shared living space (Alex)	2.28 (0.77) ^a^	2.23 (0.75) ^a^	2.22 (0.69) ^a^	2.38 (0.85) ^ab^
**Cognitions total scores**	3.35 (0.79)	3.27 (0.79)	3.32 (0.77)	3.46 (0.80) ^c^
Group project (Kyle)	3.14 (0.75)	3.38 (0.75)	2.92 (0.67)	3.12 (0.75)
Club meeting (Tim)	3.40 (0.80) ^a^	3.07 (0.80) ^a^	3.49 (0.73) ^a^	3.63 (0.76) ^a^
Shared living space (Alex)	3.52 (0.78) ^ab^	3.37 (0.79) ^b^	3.54 (0.77) ^a^	3.64 (0.77) ^a^

All means were calculated prior to multiple imputations; thus, sample sizes varied slightly by variable

M: means; SD: Standard Deviation; MAS: Multidimensional Attitudes Scale, HFA: High-functioning Autism

a Significantly different from group project vignette (*p* < 0.05)

b Significantly different from club meeting vignette (*p* < 0.05)

c Significantly different from the HFA label group (*p* < 0.05)

**Fig. 1:**
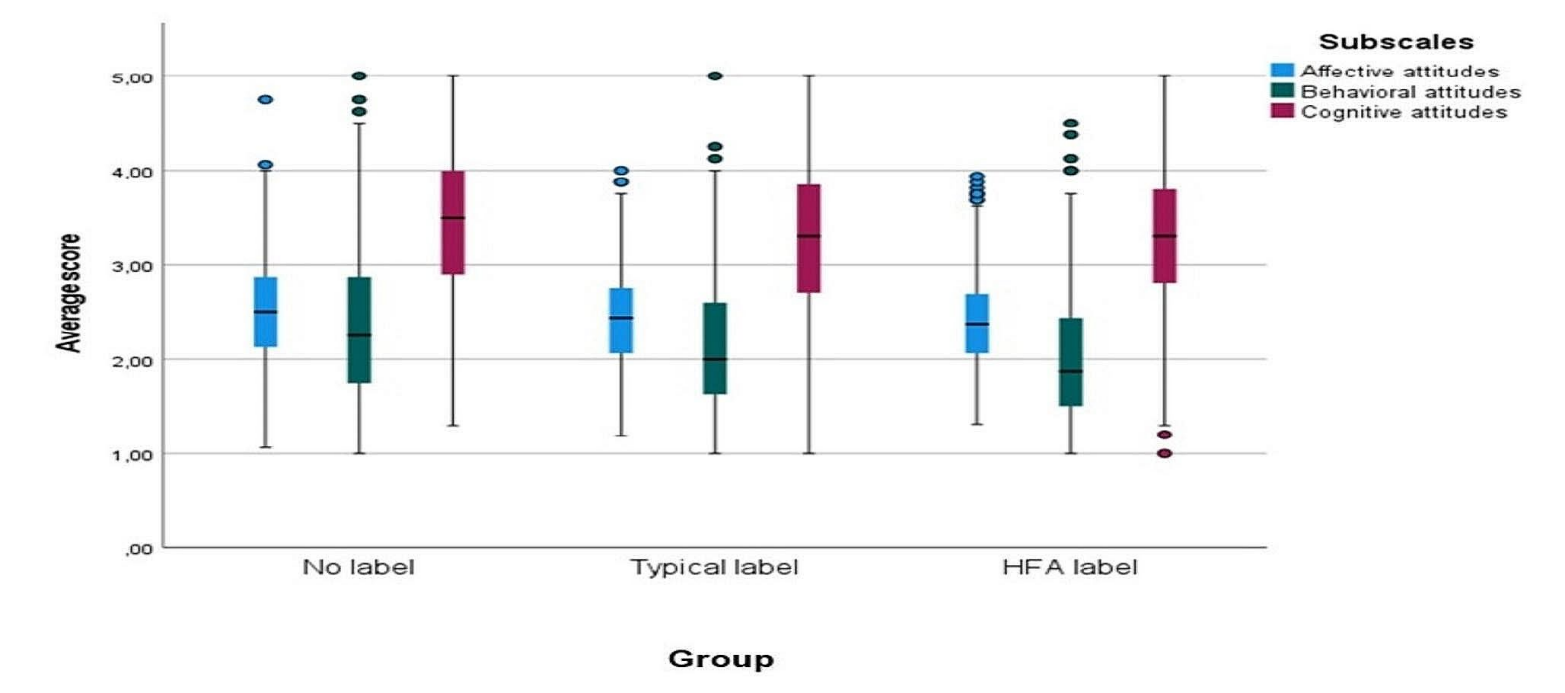
Boxplot of the average score, for the 3 groups and the 3 vignettes of interest. Although the interaction between the group and the vignette was found to be significant (*p* < 0.001), minimal effects are shown for the vignette’s differences between the different groups

**Fig. 2:**
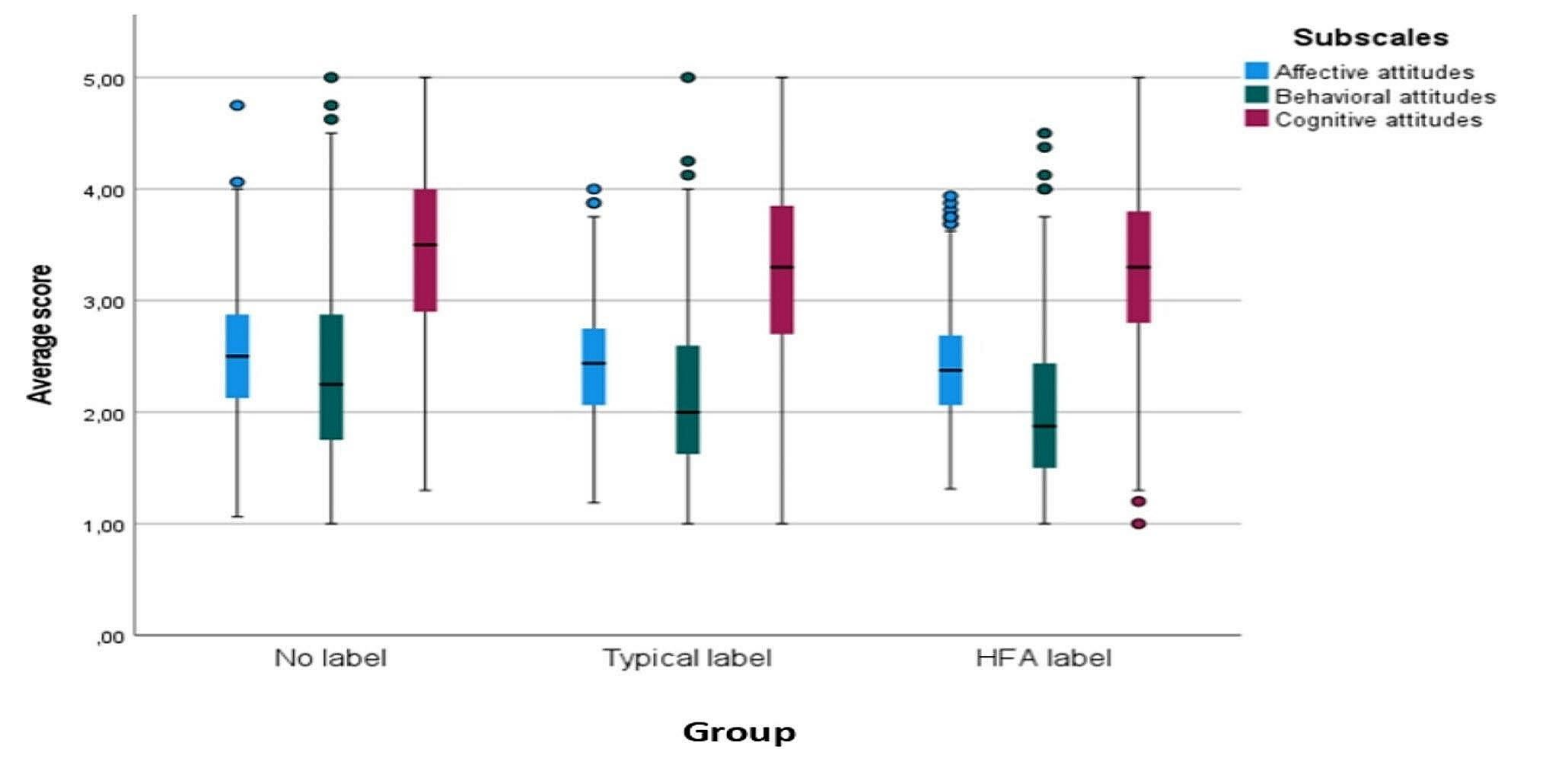
Boxplot of the average score, for the 3 groups and the 3 subscales of interest. Although the interaction between the group and the subscale was found to be significant (*p* = 0.002), minimal effects are shown for the subscale’s differences between the different groups

The interaction between the group and the vignette was found to be significant (*p* < 0.001) but the generalized Eta squared values (*n*^*2*^ = 0.009) showed minimal effect (Fig. 1) and similar results came for the interaction between group and subscale (*p* < 0.001, *n*^*2*^ = 0.002) (Fig. 2) permitting us to average subscale scores across the three vignette characters for the main hierarchical regression analysis.

## The Effect of the Label, Familiarity with ASD, and Gender on Attitudes Toward ASD

The first hierarchical regression model examined the effect of group label on affective attitudes controlling for gender and familiarity with an ASD person (see Table 4). Students in the HFA label group reported significantly more positive affective attitudes than students in the no-label group, when controlling for gender and familiarity with an ASD person (*b* = 0.115, *p* = 0.001); the comparison between the HFA label group and the Typical label group was not significant. A significant association was not observed between familiarity with an ASD person and affective attitudes and the same was true for gender.

**Table 4:** Attitudes by study group, familiarity with ASD, and gender

	Affective attitudes			Behavioral attitudes			Cognitive attitudes		
	Coef.	SE	*p*-value	Coef.	SE	*p*-value	Coef.	SE	*p*-value
***Step 1***									
Condition (reference = HFA label)									
Typical label	0.026	0.036	0.459	0.108	0.049	0.028	0.042	0.053	0.429
No label	0.116	0.036	0.001	0.299	0.049	< 0.001	0.19	0.053	< 0.001
	*ΔR*^*2*^ *= 0.009; p-value =* ***0.003***			*ΔR*^*2*^ *= 0.028; p-value <* ***0.001***			*ΔR*^*2*^ *= 0.011; p-value <* ***0.001***		
***Step 2***									
Condition (reference = HFA label)									
Typical label	0.027	0.036	0.454	0.111	0.049	0.025	0.045	0.053	0.398
No label	0.114	0.036	0.001	0.302	0.05	< 0.001	0.195	0.053	< 0.001
Relationship with ASD (reference = No)									
I have a friend	-0.070	0.041	0.086	-0.121	0.056	0.031	-0.101	0.06	0.095
I have a relative	0.017	0.098	0.860	-0.133	0.136	0.329	-0.181	0.146	0.215
	*ΔR*^*2*^ *= 0.002; p-value = 0.219*			*ΔR*^*2*^ *= 0.004; p-value = 0.068*			*ΔR*^*2*^ *= 0.003; p-value = 0.129*		
***Step 3***									
Condition (reference = HFA label)									
Typical label	0.027	0.036	0.443	0.11	0.049	0.026	0.046	0.053	0.38
No label	0.115	0.036	0.001	0.301	0.05	< 0.001	0.198	0.053	< 0.001
Relationship with ASD (reference = No)									
I have a friend	-0.074	0.041	0.068	-0.115	0.056	0.041	-0.113	0.06	0.064
I have a relative	0.022	0.098	0.822	-0.139	0.136	0.305	-0.17	0.146	0.244
Gender (reference = female)									
Male	-0.077	0.044	0.078	0.107	0.06	0.076	-0.186	0.065	0.004
	*ΔR*^*2*^ *= 0.002; p-value = 0.078*			*ΔR*^*2*^ *= 0.002; p-value = 0.076*			*ΔR*^*2*^ *= 0.006; p-value =* ***0.004***		

Coef: coefficient; SE: Standard Error; HFA: High-functioning Autism; ΔR2: delta R squares or semi-partial correlation coefficients

The second hierarchical regression model examined the effect of the label group on behavioral attitudes (see Table 4). Students in the HFA label group showed significantly more positive behavioral attitudes than students in the no-label group, when controlling for familiarity with an ASD person and gender (*b* = 0.301, *p* < 0.001); the comparison between the HFA label group and Typical label group was also not significant and in this MAS subscale. There was not a significant association between gender and behavioral attitudes or familiarity with an ASD person and behavioral attitudes.

The third and final regression model examined the effect of group label on cognitive attitudes (see Table 4). Students in the HFA label group reported significantly more positive cognitive attitudes than university students in the no-label group when controlling for gender and relationship with an ASD person (*b* = 0.198, *p* < 0.001); the HFA group and Typical label group did not differ significantly. Male students reported significantly more positive cognitive attitudes than female students (*b* = 0.065, *p* = 0.004). Entering the gender on the third step of the hierarchical regression model resulted in a statistically significant decrease over group label in explained variance (*ΔR*^*2*^ = 0.006; *p* = 0.004).

## Discussion

The current study aimed to translate and adapt the MAS scale for postsecondary education (Matthews et al., 2015) in Greek to evaluate attitudes towards ASD individuals and assess its psychometric properties, as well as to investigate the perceptions and attitudes of Greek undergraduate healthcare students about their cognitively able fellow students with ASD. The results indicated that the adapted Greek MAS scale for ASD presents excellent internal consistency and good concurrent validity and can be used to evaluate attitudes toward ASD students. The statistical analysis also showed that the group label significantly affected all three subscales of MAS (affective, behavioral, and cognitive), while familiarity with ASD did not affect any of them. Male gender significantly affected only the cognitive subscale, but not the affective or behavioral subscales.

In Greece, no previous study has ever examined attitudes towards ASD university students, whereas a recent law (Law 6069 (issue B) /28-11-2022) entitles ASD individuals to enter university without taking exams; thus, making the investigation of attitudes toward ASD fellow students of major importance since it can help in the draw line of policymakers, impacting on the successful inclusion of ASD students in the university campus. To investigate this issue, the current study utilized an indirect experimental design where the attitudes toward hypothetical individuals with autistic behaviors were examined without the threat of the social desirability bias (Antonak & Livneh, 2000).

### Psychometric Properties of the Adapted Greek MAS Scale for ASD

The adapted Greek MAS scale for ASD presented excellent internal consistency with Cronbach’s alpha coefficients exceeding 0.80. Interestingly enough, when the total scores of the MAS subscales were compared in the total sample of participants, the behavioral subscale presented significantly lower mean scores in comparison with the affects and cognitions subscales, indicating that the behavior of students revealed less negative attitudes towards peers with ASD than affects and cognitions. Thus, it seems that peers do not behave (or admit to behave) based on their feelings and thoughts of discomfort with ASD fellow students, but they adapt their behavior to the social norms and expectations. To assess external validity Spearman’s correlation coefficients between MAS subscales and SATA total scores were implemented. Two factors of the adapted Greek MAS scale correlated significantly with the SATA total scores, indicating the validity of this adapted and translated MAS version. The only factor that did not correlate with SATA total scores was the cognitive subscale, the factor found to reflect the least positive attitudes. The above makes us conclude that the SATA scale may measure the more socially acceptable and desirable aspects of attitude and highlights the need to embrace a multidimensional perspective when assessing attitudes toward people with disabilities, as proposed by Antonak and Linveh (2000). These findings are similar to those of the original validation of the MAS scale (Findler et al., 2007).

## The Effect of the Label, Familiarity with ASD, and Gender on Attitudes Toward ASD

The statistical analysis revealed significant differences between the three study groups of participants and social situations. Students in the HFA label group reported more positive attitudes toward hypothetical peers with ASD compared to the no-label group in all MAS subscales. Overall, no significant difference was found between the typical label group and the HFA label group, except for the behavioral MAS subscale. The typical label group represents university students with autistic characteristics, who have chosen not to disclose their diagnosis. The above results imply that students would behave more negatively in a fellow student with ASD when they did not understand the reason for his atypical behavior. The results of the present study expand the findings of Matthews et al. (2015) and Butler and Gillis (2011) studies, which supported that the label of ASD affects positively the attitudes of university students toward their fellow students with ASD. They also indicate that the withdrawal of the diagnosis of ASD may elicit more rejecting behaviors. Our findings also highlight Butler and Gillis’s (2011) claims who suggested that the social deficits commonly observed in individuals with ASD lead to the stigmatization and rejection of individuals with ASD and not the label itself.

Moreover, the study’s participants declared generally more negative attitudes toward the social condition of sharing living space, a finding that reflects the results of Gardiner and Iarocci’s (2014) study, which revealed that college students accept more ASD peers in terms of more distant relationships. Recent research (Park et al., 2023) underlines the importance of using realistic and meaningful vignettes to capture differences in attitude for real-life situations and this was done in the present study. Additionally, male students had more positive perceptions across study groups. Our finding is consistent with what is reported in other studies, which support that male students hold more positive attitudes towards vignette characters with autistic characteristics (Matthews et al., 2015). In Matthews et al. (2015) study, male participants demonstrated significantly more positive affective, cognitive, and behavioral attitudes toward hypothetical peers with ASD. Our findings may indicate that male students cognitively can associate more with atypical behaviors than female students. Finally, regression analysis revealed that previous social contact with an individual with ASD did not significantly predict more positive attitudes toward the hypothetical vignette character with ASD, a finding that corroborates the results of Gardiner and Iarocci’s (2014) study, claiming that the quality of direct contact has a significant impact on attitudes toward people with ASD and not just the experience of the direct contact.

### Implications, Limitations, and Future Directions

The current study investigated the impact of the label on peer attitudes toward hypothetical university students’ characters with ASD. The findings demonstrated that the knowledge of the ASD diagnosis affected significantly and positively the attitudes toward hypothetical individuals with ASD. Furthermore, the results revealed that only ASD behavior is associated with negative attitudes, indicating that individuals with ASD are likely to experience rejection and stigmatization. Moreover, neurotypical participants declared more positive attitudes toward individuals with ASD, when they engaged with them in more distant social conditions and the previous contact with people with ASD did not seem to affect significantly attitudes toward this group of fellow students. These findings have significant implications for both public policy and the treatment of individuals with ASD. Regarding public policy, the present study underlines the need for university supporting services to facilitate the transition of freshmen students with ASD into university campuses, providing simple and easy-to-access information about their role, which may lead possible ASD students to disclose their diagnosis and seek support and counseling services more easily when needed. Overall, our results point out that inclusion of ASD persons cannot be achieved by changing only one dimension of attitudes e.g. behavior. In real-life situations, ASD persons may be able to perceive the dissonance between behavior and thoughts and feelings that menace authentic interactions, thus evoking feelings of rejection and stigmatization. The development of public ASD awareness programs that relate to all the components of attitudes might result in increasing peers’ understanding of ASD characteristics and lead peers to adopt less stigmatizing views towards social-communicative ASD deficits. Acceptance and positive change toward ASD persons will be achieved only when ASD persons will routinely be “seen” and served by universities.

Applications of these results are restricted to the extent that the individual with ASD was labeled as “high-functioning”, stating not only that is of above-average intelligence but also implying that is a “typical” student (Matthews et al., 2015). Our findings may differ substantially if the label “high-functioning” has not been used. The current study is the first one that examined Greek university students’ attitudes toward fellow students with ASD, exploring the impact of ASD label, familiarity with ASD, and gender on their attitudes. More research is needed to explore this issue to a greater extent, investigating which specific autistic behaviors lead to stigmatization and how quality integration groups and autism awareness training programs can modulate peer acceptance.

## References

[CR1] American Psychiatric Association (2013). *Diagnostic and statistical manual of mental disorders* (5th edition).

[CR2] Antonak, R. F., & Livneh, H. (2000). Measurement of attitudes towards persons with disabilities. *Disability and Rehabilitation: An International Multidisciplinary Journal*, *22*(5), 211–224. 10.1080/096382800296782. 10.1080/096382800296782 10813560

[CR3] Bakker, T., Krabbendam, L., Bhulai, S., Meeter, M., & Begeer, S. (2023). Predicting academic success of autistic students in higher education. *Autism*, *27*(6), 1803–1816. 10.1177/13623613221146439. 36602222 10.1177/13623613221146439 PMC10374996

[CR4] Bania, T. A., Antoniou, A. S., Theodoritsi, M., Theodoritsi, I., Charitaki, G., & Billis, E. (2021). The Interaction with Disabled persons Scale: Translation and cross-cultural validation into Greek. *Disability and Rehabilitation*, *43*(7), 988–995. 10.1080/09638288.2019.1643420. 31340137 10.1080/09638288.2019.1643420

[CR5] Butler, R. C., & Gillis, J. M. (2011). The impact of labels and behaviors on the stigmatization of adults with Asperger’s disorder. *Journal of Autism and Developmental Disorders*, *41*(6), 741–749. 10.1007/s10803-010-1093-9. 20811769 10.1007/s10803-010-1093-9

[CR6] Cai, R. Y., & Richdale, A. L. (2016). Educational experiences and needs of higher education students with Autism Spectrum Disorder. *Journal of Autism and Developmental Disorders*, *46*(1), 31–41. 10.1007/s10803-015-2535-1. 26216381 10.1007/s10803-015-2535-1

[CR7] Dachez, J., Ndobo, A., & Ameline, A. (2015). French validation of the multidimensional attitude scale toward persons with disabilities (MAS): The case of attitudes toward Autism and their moderating factors. *Journal of Autism and Developmental Disorders*, *45*(8), 2508–2518. 10.1007/s10803-015-2417-6. 25788215 10.1007/s10803-015-2417-6

[CR19] Determining the services, method, and procedure for ascertaining severe diseases of candidates for admission to higher education (2022). 6069 B 61661.

[CR8] Findler, L., Vilchinsky, N., & Werner, S. (2007). The multidimensional attitudes Scale toward persons with disabilities (MAS): Construction and validation. *Rehabilitation Counseling Bulletin*, *50*(3), 166–176. 10.1177/00343552070500030401.

[CR9] Flood, N. L., Bulgrin, A., & Morgan, L. B. (2013). Piecing together the puzzle: Development of the Societal attitudes towards Autism (SATA) scale. *Journal of Research in Special Educational Needs*, *13*, 121–128. 10.1111/j.1471-3802.2011.01224.x.

[CR10] Gardiner, E., & Iarocci, G. (2014). Students with autism spectrum disorder in the university context: peer acceptance predicts intention to volunteer. *Journal of Autism and Developmental Disorders, 44*(5), 1008-17. 10.1007/s10803-013-1950-4. PMID: 24077739. 10.1007/s10803-013-1950-4 24077739

[CR11] Govina, O., Polikandrioti, M., Vasilopoulos, G., Adamakidou, T., Plakas, S., Kalemikerakis, I., Galanis, P., & Fouka, G. (2020). Validation with nursing students of the Greek version of the Multidimensional attitudes Scale (MAS) towards people with disabilities. *Archives of Hellenic Medicine*, *37*(4), 521–528.

[CR12] Gurbuz, E., Hanley, M., & Riby, D. M. (2019). University students with autism: The Social and Academic experiences of University in the UK. *Journal of Autism and Developmental Disorders*, *49*(2), 617–631. 10.1007/s10803-018-3741-4. 30173311 10.1007/s10803-018-3741-4 PMC6373295

[CR13] IBM Corp Released 2021. IBM SPSS statistics for Windows, Version 28.0. Armonk, NY: IBM Corp.

[CR14] Karola, D., Julie-Ann, J., & Lyn, M. (2016). School’s out forever: Postsecondary educational trajectories of students with autism. *Australian Psychologist*, *51*(4), 304–315. 10.1111/ap.12228.

[CR15] Kouznetsov, R., Angelopoulos, P., Moulinos, S., Dimakos, I., Gourzis, P., & Jelastopulu, E. (2023). Epidemiological Study of Autism Spectrum Disorders in Greece for 2021: Nationwide Prevalence in 2–17-Year-old children and Regional disparities. *Journal of Clinical Medicine*, *12*(7), 2510. 10.3390/jcm12072510. 37048594 10.3390/jcm12072510 PMC10095433

[CR16] Lei, J., Calley, S., Brosnan, M., Ashwin, C., & Russell, A. (2020). Evaluation of a transition to University Programme for students with Autism Spectrum Disorder. *Journal of Autism and Developmental Disorders*, *50*(7), 2397–2411. 10.1007/s10803-018-3776-6. 30315485 10.1007/s10803-018-3776-6 PMC7308263

[CR17] Lu, M., Pang, F., & Luo, J. (2020). Chinese validation of the Multidimensional attitude scale toward persons with disabilities (MAS): Attitudes toward Autism Spectrum disorders. *Journal of Autism and Developmental Disorders*, *50*, 3777–3789. 10.1007/s10803-020-04435-1. 32124142 10.1007/s10803-020-04435-1

[CR18] Matthews, N. L., Ly, A. R., & Goldberg, W. A. (2015). College students’ perceptions of peers with autism spectrum disorder. *Journal of Autism and Developmental Disorders, 45*(1), 90 – 9. 10.1007/s10803-014-2195-6. PMID: 25070469. 10.1007/s10803-014-2195-6 25070469

[CR20] Obeid, R., Daou, N., DeNigris, D., Shane-Simpson, C., Brooks, J. P., & Gillespie-Lynch, K. (2015). A cross-cultural comparison of knowledge and Stigma Associated with Autism Spectrum Disorder among College students in Lebanon and the United States. *Journal of Autism and Developmental Disorders*, *45*, 3520–3536. 10.1007/s10803-015-2499-1. 26084712 10.1007/s10803-015-2499-1

[CR21] Papadopoulos, D. (2021). Mothers’ experiences and challenges raising a child with Autism Spectrum disorder: A qualitative study. *Brain Sciences*, *11*(3), 309. 10.3390/brainsci11030309. 33801233 10.3390/brainsci11030309 PMC8001702

[CR22] Papadopoulos, A., Tafiadis, D., Tsapara, A., Skapinakis, P., Tzoufi, M., & Siafaka, V. (2022). Validation of the Greek version of the Affiliate Stigma Scale among mothers of children with autism spectrum disorder. *BJPsych Open*, *20*(8(1)), e30. 10.1192/bjo.2021.1083. 10.1192/bjo.2021.1083 PMC8811780 35045904

[CR23] Park, J., Levine, A., Kuo, H. J., Lee, B., & Beymer, P. N. (2023). Validation of the multiple disability multidimensional attitudes scale toward persons with disabilities. *Rehabilitation Psychology*, *68*(2), 194–203. 10.1037/rep0000494Epub 2023 Apr 6. PMID: 37023288. 37023288 10.1037/rep0000494

[CR24] Petcu, S. D., Zhang, D., & Li, Y. F. (2021). Students with Autism Spectrum disorders and their first-year College experiences. *International Journal of Environmental Research and Public Health*, *18*(22), 11822. 10.3390/ijerph182211822PMID: 34831577; PMCID: PMC8622457. 34831577 10.3390/ijerph182211822 PMC8622457

[CR26] Raue, K., & Lewis, L. (2011). Students With Disabilities at Degree-Granting Postsecondary Institutions (NCES 2011–018). U.S. Department of Education, National Center for Education Statistics. Washington, DC: U.S. Government Printing Office.

[CR25] R Core Team (2021). R: A language and environment for statistical computing. R Foundation for Statistical Computing, Vienna, Austria. URL https://www.R-project.org/.

[CR27] RStudio (2020). PBC, Boston, MA URL http://www.rstudio.com/.

[CR28] Rubin, D. B. (1987). *Multiple imputation for nonresponse in surveys*. Wiley.

[CR38] Robine, J. & Jagger, C. (2003). Translation & linguistic evaluation protocol & supporting material. *Geneva, Switzerland: WHO/UNESCAP Project on Health and Disability Statistics*. Wiley.

[CR39] SPSS v. 28 the following (IBM Corp. Released 2021. IBM SPSS Statistics for Windows, Version 28.0. Armonk, NY: IBM Corp.).

[CR29] Thomaidis, L., Mavroeidi, N., Richardson, C., Choleva, A., Damianos, G., Bolias, K., & Tsolia, M. (2020). Autism Spectrum disorders in Greece: Nationwide Prevalence in 10–11 year-old children and Regional disparities. *Journal of Clinical Medicine*, *9*(7), 2163. 10.3390/jcm9072163. 32650567 10.3390/jcm9072163 PMC7408756

[CR30] Tipton, L. A., & Blacher, J. (2014). Brief report: Autism awareness: Views from a campus community. *Journal of Autism and Developmental Disorders*, *44*(2), 477–483. 10.1007/s10803-013-1893-9. 23881093 10.1007/s10803-013-1893-9

[CR31] Tsujita, M., Ban, M., & Kumagaya, S. I. (2021). The Japanese Multidimensional attitudes Scale toward persons with autism spectrum disorders. *Japanese Psychological Research*, *63*, 12939. 10.1111/jpr.12298.

[CR32] Van Hees, V., Moyson, T., & Roeyers, H. (2015). Higher education experiences of students with autism spectrum disorder: Challenges, benefits, and support needs. *Journal of Autism and Developmental Disorders*, *45*(6), 1673–1688. 10.1007/s10803-014-2324-2. 25448918 10.1007/s10803-014-2324-2

[CR37] Vanbergeijk, E., Klin, A., & Volmar, F. (2008). Supporting more able students on the autism spectrum: College and Beyond. *Journal of Autism and Developmental Disorders*, *38*(7), 1359–1370. 10.1007/s10803-007-0524-8. 10.1007/s10803-007-0524-8 18172747

[CR33] Veroni, E. (2019). The Social Stigma and the challenges of raising a child with Autism Spectrum disorders (ASD) in Greece. *Exchanges: The Interdisciplinary Research Journal*, *6*(2), 1–29. 10.31273/eirj.v6i2.200.

[CR34] White, D., Hillier, A., Frye, A., & Makrez, E. (2016). College Students’ knowledge and attitudes towards students on the Autism Spectrum. *Journal of Autism and Developmental Disorders*, *49*, 2699–2705. 10.1007/s10803-016-2818. 10.1007/s10803-016-2818-1 27230760

[CR35] Zarokanellou, V., Gryparis, A., Papatheodorou, P., Tatsis, G., Tafiadis, D., Papadopoulos, A., Voniati, L., & Siafaka, V. (2023). Societal attitudes towards autism (SATA): Validation of the Greek Version in the General Population. *Journal of Autism and Developmental Disorders*. 10.1007/s10803-022-05842-2. Epub ahead of print. PMID: 36626003. 36626003 10.1007/s10803-022-05842-2 PMC10981628

[CR36] Zeidan, J., Fombonne, E., Scorah, J., Ibrahim, A., Durkin, M. S., Saxena, S., Yusuf, A., Shih, A., & Elsabbagh, M. (2022). Global prevalence of autism: A systematic review update. *Autism Research*, *15*(5), 778–790. 10.1002/aur.2696. 35238171 10.1002/aur.2696 PMC9310578

